# Contextual attributes to promote positive social interdependence in problem-based learning: a focus group study

**DOI:** 10.1186/s12909-021-02667-y

**Published:** 2021-04-21

**Authors:** Ikuo Shimizu, Yasushi Matsuyama, Robbert Duvivier, Cees van der Vleuten

**Affiliations:** 1 Center for Medical Education and Clinical Training, Shinshu University, 3-1-1 Asahi, Matsumoto, 3908621 Japan; 2 Medical Education Centre, Jichi Medical University, 3311-1 Yakushiji, Shimotsuke-shi, Tochigi Japan; 3 Center for Educational Development and Research in Health Sciences (CEDAR), University Medical Center Groningen, Antonius Deusinglaan 1, 9713 AV Groningen, The Netherlands; 4 Department of Educational Development and Research, Faculty of Health, Medicine and Life Sciences, Maastricht University, Universiteitssingel 60, 6229 ER Maastricht, The Netherlands

**Keywords:** Collaborative learning, Health professions education, Problem-based learning, Social interdependence theory, Qualitative research

## Abstract

**Background:**

Problem-based learning (PBL) is classified as a collaborative learning approach, wherein students learn while contributing meaning to experiences and interactions with others. An important theoretical fundament of PBL is social interdependence theory (SIT) because positive social interdependence within a group has been found to be key to better learning performance and future attitudes towards team practice. However, most previous studies in health professions education focused on cognitive outcomes, and few studies have focused on collaborative behaviors in PBL groups. The lack of this empirical insight makes implementation of PBL difficult, especially in contexts where there is limited experience with collaborative learning. Therefore, the aim of this study was to elucidate what promotes or hinders positive social interdependence and how the attributes work during PBL.

**Methods:**

We conducted four focus groups among clinical year medical students (*n* = 26) who participated in PBL tutorials in the formal curriculum. We asked semi-structured questions that corresponded with the overall concept of SIT. We analyzed the transcript using constructivist grounded theory and developed a model to explain contextual attributes that promote or hinder positive social interdependence in PBL.

**Results:**

Two contextual attributes of “academic inquisition” and “desire for efficiency” affect social interdependence among a student group in PBL. Academic inquisition is students’ desire to engage in their academic learning, and desire for efficiency is students’ attitude toward learning as an imposed duty and desire to complete it as quickly as possible. These attributes are initially mutually conflicting and constructing social interdependence through multiple steps including inquisition from a case, seeking efficient work, sharing interest in problem solving, expecting mutual contributions, and complementing learning objectives.

**Conclusion:**

These findings will contribute to understanding collaborative learning environments in PBL and may help explain contexts where PBL is less successful. The model can also be used as a tool to support innovation of PBL as collaborative learning.

**Supplementary Information:**

The online version contains supplementary material available at 10.1186/s12909-021-02667-y.

## Introduction

Problem-based learning (PBL) has been utilized in global professional health education for more than 50 years. It is classified as a collaborative learning approach, wherein students learn while contributing meaning to experiences and interactions with others [1, 2]. The quality of discussions within tutorial groups make significant contributions to the success of PBL [3]. In group discussions, for learning outcomes to be achieved, there must be group dynamics that involve participant cooperation.

Since group dynamics are essential for small group discussion, social interdependence theory (SIT) has been applied to PBL as one of theoretical frameworks” [4]. In this theory, social interdependence exists when the outcomes of individuals are affected by their own and others’ actions, and the process to structure positive and negative interdependence is divided into three categories: outcome, means, and boundary [5]. Outcome interdependence is defined as orientation toward goals and rewards. Means interdependence includes resources, roles, and task interdependence. Resources are used among group members, some of which are utilized as joint property. Roles are assigned to group participants, such as readers, recorders, summarizers, and encouragers. Task interdependence can be created when the group members come to mutual agreement regarding how to divide and assign the tasks, making each group member responsible for their learning objectives. This leads the learning group to be more productive. Boundary interdependence is based on abrupt discontinuities among individuals, and thus includes identity and environment (such as a working area) [4].

There are positive (the actions to promote the achievement of joint goals) and negative (the actions to obstruct the achievement of each other’s goals) types of social interdependence. Positive interdependence is a key for successful collaborative learning [6] because positive interdependent cooperation does results in more frequent use of higher-level reasoning, more interpersonal relationships, and greater social support [5]. Furthermore, social interdependence is important in health professionals so that they construct relationships between intra- and interprofessional care providers, trainees and trainers and patients [7]. This is a reason why some medical educators [8, 9] wanted to cultivate positive social interdependent attitudes among learners through PBL.

However, we have little knowledge regarding what promotes or hinders positive social interdependence in PBL. Most previous PBL studies paid attention to aspects of cognitive outcomes, and only few studies have focused on collaborative behaviors in PBL groups [10]. Although the results of PBL can be observed through behavioral and psychological changes [11–14], it is unclear which details in PBL augment social interdependence. The lack of this explanation makes it difficult to correctly implement PBL and the challenge can be seen in different parts of the globe, as for example, Asia [8]. In a Japanese medical school, Oda and Koizumi [15] faced difficulties such as superficial discussion and significant differences in learning attitudes among students, as well as limitations in tutors’ skills. Khoo [16] described how Asian contextual characteristics might be incompatible with discussions in PBL. It might be because the existing educational systems and environments were incompatible with PBL [17]. However, they have not been able to explain why the educational system and environment were not compatible with the collaborative characteristics in PBL because the promoting and inhibiting factors of social interdependence in the PBL groups have not been sufficiently clarified from the perspective of SIT. If we can explain collaborative behavior in PBL using SIT, we will be able to analyze the functioning of PBL in various contexts and therefore propose methods to optimize PBL in individual contexts. The aim of this study, therefore, was to elucidate what promotes or hinders positive social interdependence and how social interdependence functions within the PBL group.

## Methods

This study employed a constructivist grounded theory approach [17] to elucidate social interdependence that students had cultivated during PBL, based on an interpretivist paradigm that reality is context-dependent and that multiple interpretations can be constructed among people [18].

Participants included fourth-year medical students of the six-year undergraduate medical curriculum in Shinshu University, Japan. The hybrid curriculum included lectures and collaborative learning opportunities followed by the PBL. The students had completed a set of PBL tutorials (comprised of two tutorial sessions) during the internal medicine II clinical rotation and participated in targeted PBL as a part of the formal curriculum. The PBL covered clinical reasoning of hematology cases and was conducted in concordance with the original seven-step approach [1], as accurately as possible. One author (IS) served as a tutor to avoid wide variance in tutoring skills during the discussions. Since there could be criticism that Asian faculty have conducted PBL differently under the teacher-centered and examination-based learning culture [8, 19], the tutor has understood the notion and tried not to let the power difference affect the discussions and reflect his facilitation. The PBL tutorial is not related to the grading of students; summative assessment during the internal medicine rotation was workplace-based with clinician-educators, then the students will take summative graduation test after completing all of the clinical clerkship rotations. The PBL tutorial in this study was only used for formative assessment.

Data collection and analysis then occurred in an iterative fashion. We asked students to participate in the research before initiating PBL and conducted focus groups comprised of students who were accepted as study participants. We used theoretical sampling [20] based on an assumption that some students had favorable perception on collaborative learning while others may not. We formed a focus group with the same members as the respective PBL groups because we wanted to stimulate them to recall the interactions between the participants during their discussions [21].

A semi-structured focus group was conducted after the entire PBL sessions were completed. Informed consent was obtained as declared in the ethical consideration. During the focus groups, the primary researcher (IS) asked participants questions (see Additional file 1) and recorded all conversations during the sessions. He was exempted from summative assessment of the clerkship to allay the concern that participants’ comments during responses might affect their assessment. An administrative clerk helped organizing and assisted recording the focus groups, and another researcher (YM) checked the recorded data before coding for triangulation.

Questions used in focus groups were formulated to correspond to the overall concept of social interdependence in collaborative learning and the three components of SIT [4]. The first question cued participants to recall words and actions that helped the group, or conversely, that helped them study independently. The focus group continued with a discussion that followed the questions shown in the interview guide (see Additional file 1).

The interview guide was periodically revised in light of the developing analytical process by researchers (IS and YM). Iterative comparison was conducted by comparing the data with the previous group until saturation was reached [17, 22]. We initially enrolled students who were interested in giving an active opinion about the group discussion, and conducted three focus groups during the 2017–2018 clinical clerkship program. Then we conducted one more focus group to obtain more rigor from members of a PBL group who perceived their collaboration did not work well during their discussion in 2019, in case the students who voluntarily participated in the discussion might make good use of the group discussion. Every focus group took 45–60 min. Ultimately, 26 students were enrolled until saturation, comprising four focus groups in total. The participants consisted of 17 males and 9 females, which showed a comparable male/female ratio to this medical school (2.07 in 2020). Median age was 23 (range: 21–36). All recorded interviews were subsequently transcribed by a research assistant service.

Coding and categorization with theoretical sampling and repetitive comparison were conducted as the processes of constructivist grounded theory to elucidate the contextual attributes that may promote or hamper social interdependence in the PBL. We referred to items in the social interdependence in collaborative learning scale [23] for the coding process because it includes several behaviors congruent with the three components of SIT. We chose open and axial coding based on the definition of social interdependence [4] without using the framework or three components of SIT because there has been no prior literature to explain the specific processes in line with them. Coding as well as inductive categorization, were conducted by the two authors (IS and YM) in Japanese. They initially read the transcript and coded individually for triangulation, and then reviewed and matched them together. This process was conducted iteratively. Since the first author was also a tutor, he took a memo for his reflection on the process and the researcher’s role and influence after each interview and when reading the transcript to ensure reflexivity. Representative speech-supporting codes were translated once into English in the selective coding phase and a proof reading service then confirmed translation. The other authors (RD and CvdV) contributed to develop the manuscript through discussion. We used Microsoft Excel throughout the coding process.

## Results

Through the analysis of the audio transcripts, we identified two different types of contextual attributes that affect the fundamental concept of social interdependence. The first attribute refers to students’ interest in engaging and deepening their academic learning. The other attribute refers to students’ attitude that they regard learning contents as imposed duties, and want to complete them as minimal time and effort as possible. These are initially perceived as mutually conflicting concepts in constructing interactions. Thus we named “academic inquisition” and “desire for efficiency” as two contextual attributes that promote or hinder positive interdependence of students in PBL, and regarded as the key themes in the result of the research.

**Fig. 1:**
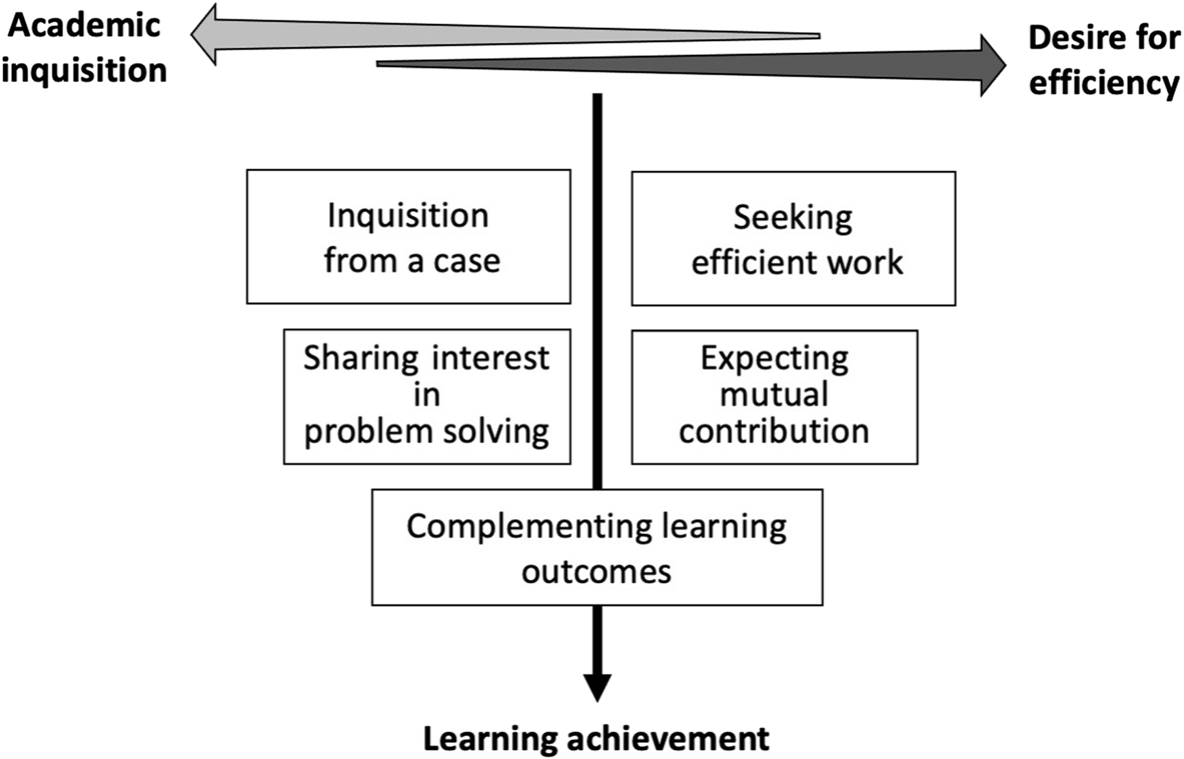
The two contextual attributes that promote or hinder positive interdependence of students in PBL, and the five steps that the contextual attributes affect social interdependence during the discussion

Figure 1 depicts the five steps that these contextual attributes above (academic inquisition and desire for work efficiency) affect social interdependence during the PBL sesions. In this model, students’ inquisition from a case was provoked, and they felt compelled to proceed with their learning. Simultaneously, they regarded PBL as one of the duties and wanted to seek an efficient work process to complete the work as quickly as possible. As a result, they were willing to share their academic problems and work together to solve the problems even though they were not familiar with each other. They also expected each other’s contributions to increase efficiency. Eventually, they considered their sharing and contributions resulted in the socially interdependent behavior of complementing the learning outcomes.

We categorized the inquisition from a case and seeking efficient work steps as attitudes that prioritize academic inquisition and desire for efficiency, respectively. The subsequent sharing interest in problem solving and expecting mutual contributions were rooted in these attributes, respectively, but were created through expectations of others’ behavior as PBL processes progressed. The final behavior resulted in complementing learning outcomes. Although we also observed other minor items which emerged less frequently, they were considered either subsets of the steps or unrelated to our aim (e.g. comments on clinical clerkship).

The following sections explain the five steps to establish positive social interdependence during PBL. Representative quotes are presented below to exemplify each step.

### Inquisition from the case

The students were initially motivated by what they found interesting in their cases that drove their learning process. They were aware of the significance of sharing their problems with other students, who might have different interests from their own. These behaviors were categorized as inquisition from the case.

*My initial purpose of PBL was supposed to be to study myself, but when I started PBL and started to work on case studies, I felt emotions like "this is really interesting" and "I want to share my emotions". As soon as you are interested in the case, you want to tell others about it.*

*As I do, each person has their own problems that they find from this case, so by being aware that others have other perspectives on things that you haven't gone to see, you will be able to pay attention to them.*

### Sharing interest in problem solving

The students believed that they needed to share their own insights, which was derived from their inquisition. They also wanted to share questions related to achieving academic goals as they believed that this would help create PBL. These behaviors were categorized as sharing interest in problem solving.

*I want to share my opinions because I expect groups to make me aware of points I don't notice, and that's what I'm looking for in a group. In addition, I think it's easier to move on to the next step if we see the same things and build consensus on moving forward as group work, rather than having it all to ourselves.*

*I didn't care that the patient in my case today has hypernatremia. So if you don't speak up with the assumption that everyone will understand the problem, it's a risk to the group. That (speaking up) is what I think learning in PBL is all about.*

### Seeking efficient work

While the students were interested in the cases, PBL was perceived as a mandatory task to be completed as a part of the regular curriculum. Therefore, students wanted to complete it as efficiently as possible. They believed that a knowledgeable facilitator would be able to help drive efficient progress. These behaviors were categorized as seeking efficient work.

*Since it's group work within a set amount of time, I think it would be more efficient if the person with more knowledge would take the lead and have a better time to complete the tasks, which would increase the overall efficiency.*

*I thought that someone who can do better than me should do to moderate. If there is someone like that in there, I shouldn't be the one to do it. It's faster or more efficient. (What do you mean "do better" ?) Having some knowledge on the theme. We could do it more quickly if there were such a person.*

### Expecting mutual contributions

Each student tried to find interesting points in the cases and also to estimate their level of understanding regarding the topic for efficient work process. It did not matter that the students had different levels of understanding; instead, they found it important to identify those differences and contribute appropriately to the learning process so that the learning could be completed. This was referred to as expecting mutual contributions.

*I show everyone what I know. Often, others will point out things that I didn't notice or something else, so I may show them hoping that they will notice something I didn't notice.*

*One of the advantages of group learning is that you can get ideas from other people you didn't have when studying alone. For example, even if you are working as a doctor in clinical practice, the nurses will speak from a different perspective, so it's worth listening to them and expecting their opinions.*

### Complementing the learning outcomes

Finally, the students attempted to achieve more meaningful learning outcomes by complementing their academic achievements with the achievements of others. They believed this would increase their learning and improve their learning efficiency through mutual contributions. These behaviors were categorized as complementing the learning outcomes.

*(How did you decide on your academic objectives?) As all of us weren't sure about the lung images, we all agreed to do it together. But other than that, we went over each of the things we had listed and the things we wanted to look into, and then we decided on the learning objectives for each of us.*

*I'm responsible for my own learning goals because I chose them, and since there are five of us, I'm sure the other four will learn properly, and that's the brake on me. I don't want to be lazy or wonder if that's enough, but I want to prepare myself so that I don't have to be disrespectful to someone else who has been working harder than me have to be prepared for that. Motivation from the inside is important, but there is also motivation from the outside, which is created by others' presence, and I feel that this is an advantage of learning in a group.*

Conversely, there were some students who felt that they could not expect contributions from others or could not achieve more than their own learning through the group discussion. They did not establish social interdependence and were instead oriented toward individual learning. While they were interested in learning, they did not find group learning to be efficient. Others were so concerned with learning efficiency that they were distracted by the opinions of their tutors, who should have known the conclusions, rather than their own interests.

*What makes a person grow the most is when he or she is in a group. But there are times when I think I'm better off on my own than in that group. Although individual learning is the next best thing, there have been few moments when I've learned in a group where I've been able to surpass my own efforts, so it's tempting to prioritize my own learning.*

*I was inclined to discuss it, but I was too conscious of reacting to the tutor's advice. I was thinking about reaching the end the tutor had for us rather than setting our own academic goals.*

## Discussion

This study revealed contextual attributes that promote positive social interdependence during PBL and how they function. Two attributes that affect social interdependence were uncovered: academic inquisition and desire for efficiency. Then we explained the processes to work the attributes with five steps. Ultimately, these attributes culminated in the interdependent behavior of complementing each other’s learning outcomes.

PBL is said to foster positive social interdependence [24, 25]. This study revealed the process by which this occurs by analyzing a PBL program in a Japanese context. We begin by discussing whether the results are consistent with SIT as a theoretical framework and its components (outcome, means, boundary) [5]. First, the students’ academic interest in the case attracted their attention and made them seek out problems to solve. Simultaneously, the students consider solving the problems to be a task that was completed in a fixed curriculum where they were expected to finish it properly. This attitude indicates that academic inquisition and desire for efficiency in PBL facilitates positive outcome interdependence because structuring situations that support it results in increased effectiveness and productivity [26].

The students dealt with these perceptions through two measures. One was to speak up and share their academic inquiries, and the other was to seek each other’s contributions to increase efficiency. These led to a mutually complementary behavior of wanting to share their ideas and learning achievements. We considered that the processes resulted in positive means interdependence because the process includes interaction patterns through task, role, and resource [4] . Because PBL was seen as a set task in an official curriculum, the students’ willingness to complete the task may have promoted task interdependence. Then, when each student was expected to contribute to the promotion of task interdependence, the use of role interdependence was required. Some knowledgeable students and tutors were expected to have a role in making progress as well. We can observe these findings within the group discussion phase of PBL (e.g. steps 1–5 in the seven-steps approach). The remaining steps are also related because step 6 is self-study for the complemented learning objectives and, in step 7, students integrate information within the group. Thus, while we have found contextual attributes within the discussion phase of PBL in this study, they presuppose the subsequent steps of self-study and learning integration. The students purposefully acted on the social interdependence described above for the sake of academic inquiry and efficiency. These behaviors are similar to previous research on non-learning environments. Wageman [27] explains that group achievement increases cooperation while the level of perceived effort affects the quality of group performance in his research at a large corporation.

The remaining component, boundary interdependence was also observed. Students were asked to understand the differences of opinion in their groups and to contribute their diverse perspectives based on those differences. The findings we observed in this study are consistent with previous articles. For example, Torre et al. [28] claim that entitativity (the perception of a group as a single entity) is important in PBL because it affects the group’s pursuit of common goals and group decisions. In addition, they also advocate that individual responsibility plays another key role in the collaboration [28]. Because the performance of a member affects the outcome of the whole group, each member feels responsible for the performance outcome. There is a concern that “social loafing” can lead to unproductive work in the group [29]. This is unlikely if discussion is well designed to establish individual accountability and engage personal performance with group achievement, including changing the group allocation process [30] and providing the underlying ideas of PBL [31].

Research shows that PBL cultivates social interdependence and offers a partial explanation of group dynamics in general [23], such as the success of group learning [32]. However, PBL does not only occur in group work; the perceptions and work of each participant affects discussions in the curricula of undergraduate health professions. The students are forced to think about individual achievement and learning outcomes as long as they receive the high-stakes assessment of themselves such as graduation test or national license examination in the near future [33]. Since group functioning and individual contributions are difficult to separate in terms of successful learning [19], it is necessary to assess not only knowledge but also group dynamics in PBL. The results of this study will contribute to that assessment through the lens of social interdependence.

Our findings also explain one of the situations that PBL did not function as expected. A report of the such situation in Asia [8] revealed that, the students were confused by the tutor’s demand for self-directed learning, and the tutor was frustrated by the students’ inability to deepen the discussion. There has also been debate about the reasons for the outcome that PBL did not work after implementing it. While they have been discussed as independent factors, our findings explain this phenomenon as a failure to provide efficiency in PBL because individual academic inquiry was not cultivated. As some educators pointed out [14], their PBL practices were not sufficiently linked to the achievement goals in their undergraduate program. Therefore, their PBL practices failed to evoke the academic inquiry that is essential for constructing academic goals within PBL. In addition, the East Asian emphasis on the significance of high-stakes testing in learning made students more aware of operational efficiency in learning rather than academic inquisition [33]. As a result, the students expected that someone who had enough knowledge to learn would take the initiative to guide them through the process. Hence, a side effect of the stronger burden on the tutors also manifested as a relative insufficiency of the tutors’ skills [14]. Therefore, the process of exchanging and sharing ideas through discussion did not feel more efficient than independent study in the theoretical framework of social interdependence and did not create an environment for students to have constructive group discussions. Another argument about Asian students in PBL is that they typically avoid dialogues at the expense of their own interests [15]. Mutual contribution through dialogue will take a lot of time and undermines the efficiency of learning. If the tutor fails to arouse enough interest to merit discussion, the student will try to avoid dialogues and work through the discussion promptly.

Several factors inhibit the discussion phase of PBL, such as previous educational systems, tutor behavior, and assessment systems [16]. Our findings are consistent with these factors. If the existing educational system is passive in handling tasks, it must prioritize efficiency over academic inquiry. Students’ expectations of tutor behavior would also be heavily weighted toward simply providing the knowledge that is required for learning. The response to high-stakes examinations also prioritizes efficiency. Regardless of whether this phenomenon is judged a “failure,” it is an adaptation of PBL to the East Asian context [34].

Our findings can be used to improve PBL using SIT as a theoretical framework. It is necessary to strike a balance between academic inquiry and efficiency. As mentioned above, PBL practices in East Asia have overemphasized operational efficiency; therefore, instruction that can guide students to encourage academic inquiry would be useful. In the assessment, not only the acquisition of learning items, but also attitudinal items, such as outcomes and social interdependence in the means of learning, could be assessed. Since skills to promote positive social interdependence can be trained [35], any feedback provided through the lens of SIT may be useful.

This model could also be applied as an innovative tool for collaborative learning. One example is the enhancement of boundary interdependence by comparing the learning outcomes of groups, which is a strategy for creating boundary interdependence [36]. PBL is not fundamentally designed to compare learning achievement between groups, and we did not find any evidence that other groups influenced social interdependence in PBL. For example, if we could provide an opportunity for students to show their reaction towards the case beyond the group and discuss in a larger group, we could strengthen the processes of social interdependence in our model. Alternatively, future studies might include technology-enhanced learning, as technology will certainly contribute to future education [37], and online collaborative learning is becoming more popular [38]. However, problem-solving in the online environment is sometimes frustrating for students [39] since the quality of communication decreases in virtual discussions. There have been several reports on online or blended PBL, some of which succeeded in technically fostering the group process or improving the cooperation during the self-directed learning [40, 41] . We should ensure positive social interdependence as much as possible based on our findings when we conduct further online or blended PBL. For example, using chat and response tools together to encourage participants to participate in discussions will make it easier to share them and make their contributions visible to each other [42]. In addition, using a learning management system to assess understanding instead of relying on tutors [43] would complement academic inquisition. These specific methods should be explored in future research.

### Strengths and limitations

These findings might enable innovations that new intervention procedures can be suggested for tutors. Since tutor training is a crucial component of a successful PBL curriculum [44], various curricula to improve tutors’ competencies in PBL have been implemented. For example, Azer [45] suggested twelve tips, such as building trust and encouraging the bonding of group members, as well as promoting group dynamics. However, balancing academic inquisition and desire for efficiency will be required in terms of positive social interdependence, in addition to group cohesiveness. For example, encouraging professional identity formation and self-directed learning attitude [46] by self-reflection about students’ social expectations and personal identity as a future profession [47], may introduce student more academic inquisition and thus make PBL sessions more beneficial.

On the other hand, there are some limitations. First, we did not make assumptions about the cultural characteristics of the participants, which might affect the findings of our research, since we can refer the difference of social interdependence perception into rejection avoidance and harmony-seeking attitudes. According to their study, there is no difference in harmony seeking between Japan and the United States, but Japanese respondents reveal higher rejection avoidance. When the notion is transferred to this model, attitude to pursue desire for efficiency might be strengthened while sharing inquisition might be decreased.

In addition, the PBL tutor also served as the interview and analyst. It is possible that this may have had an impact on the collection and analysis of data from students. However, he was not involved in the summative assessment and he regularly reflected on the text and analysis with the other authors to reduce the impact as much as possible to ensure reflexivity of the research.

In conclusion, this study revealed that there were two contextual attributes (academic inquisition and desire for efficiency) for positive social interdependence in PBL based on analysis in an East Asian undergraduate context. In the pursuit of both academic inquiry and operational efficiency, students created a positive social interdependence that called for shared problem-solving and mutual contributions. From these findings, further analysis of the phenomena during discussions, training of tutors, and innovative learning environments are determined to be more effective in collaborative learning practices.

## Supplementary Information

**Additional file 1.** List of interview guide.

## Data Availability

The datasets used and/or analyzed during the current study are available from the corresponding author on reasonable request.

## Abbreviations

PBL: Problem-based learning
SIT: Social interdependence theory
