# Supplementary material for: Contextual attributes to promote positive social interdependence in problem-based learning: a focus group study
Source: BMC Med Educ. 2021 Apr 21;21:222. doi: 10.1186/s12909-021-02667-y (PMC8059308; doi:10.1186/s12909-021-02667-y)
Supplement: Supplementary file 1 — Additional file 1. List of interview guide. [file 12909_2021_2667_MOESM1_ESM.docx]

Article

Contextual attributes to promote positive social interdependence in problem-based learning: a focus group study

**Authors**

Ikuo Shimizu (corresponding author)

Center for Medical Education and Clinical Training, Shinshu University, Matsumoto, Japan

Address: 3-1-1 Asahi, Matsumoto, 3908621, Japan

Phone: +81 263-37-3359 E-mail: ishimizu@shinshu-u.ac.jp

Yasushi Matsuyama

Medical Education Centre, Jichi Medical University, Shimotsuke, Japan

Address: 3311-1 Yakushiji, Shimotsuke-shi, Tochigi, Japan

Phone: +81 285-58-7067 E-mail: yasushim@jichi.ac.jp

Robbert Duvivier

Center for Educational Development and Research in Health Sciences (CEDAR), University Medical Center Groningen, Groningen, The Netherlands

Address: Antonius Deusinglaan 1 9713 AV Groningen, The Netherlands

Phone: +31 503616161 E-mail: robbertduvivier@gmail.com

Cees van der Vleuten

Department of Educational Development and Research, Faculty of Health, Medicine and Life Sciences, Maastricht University, Maastricht, The Netherlands.

Universiteitssingel 60, 6229 ER Maastricht, The Netherlands

Phone: +31 43-3885725 E-mail: c.vandervleuten@maastrichtuniversity.nl

**Appendix: Interview guide**

- How did you learn in the PBL discussion?
- How did you share the aims of the discussion, your thoughts, and knowledge?
- How did you proceed with the discussion?
- How did you determine your roles? How did you use facilities, and why?
- How did you feel about yourself in the group during the discussion?
- How did you feel about other students outside this group?
- When did you feel a sense of collaboration or isolation in this PBL group, and why?
